# Sex-Specific Patient Journeys in Early Parkinson's Disease in the Netherlands

**DOI:** 10.3389/fneur.2019.00794

**Published:** 2019-07-30

**Authors:** Floris Pieter Vlaanderen, Yvonne de Man, Jesse H. Krijthe, Marit A. C. Tanke, A. S. Groenewoud, Patrick P. T. Jeurissen, Sabine Oertelt-Prigione, Marten Munneke, Bastiaan R. Bloem, Marjan J. Meinders

**Affiliations:** ^1^Radboud University Medical Center, Radboud Institute for Health Sciences, Scientific Institute for Quality of Healthcare, Nijmegen, Netherlands; ^2^Institute for Computing and Information Sciences, Radboud University, Nijmegen, Netherlands; ^3^Radboud University Medical Center, Department of Primary and Community Care, Nijmegen, Netherlands; ^4^Department of Neurology, Radboud University Medical Center, Donders Institute for Brain, Cognition and Behaviour, Nijmegen, Netherlands

**Keywords:** patient journey, Parkinson's disease, sex difference, personalized care, healthcare usage, early Parkinson's disease

## Abstract

**Objective:** To reconstruct a sex-specific patient journey for Dutch persons with Parkinson's disease (PD) during the first 5 years after diagnosis.

**Method:** We analyzed a national administrative medical claims database containing data of all patients newly diagnosed with PD between 2012 and 2016 in the Netherlands. We performed time-to-event analysis to identify the moments when patients received care from neurologists, allied healthcare therapists or general practitioners. We also extracted relevant clinical milestones: unexpected hospitalization for PD, pneumonia, orthopedic injuries, nursing home admission, and death. Using these data, we constructed the patient journey stratified for sex.

**Results:** We included claims data of 13,518 men and 8,775 women with newly diagnosed PD in the Netherlands. While we found little difference in neurologist consultations, women visited general practitioners and physiotherapists significantly earlier and more often (all *p*-values < 0.001). After 5 years, 37.9% (*n* = 3,326) of women had visited an occupational therapist and 18.5% (*n* = 1,623) a speech and language therapist at least once. This was 33.1% (*n* = 4,474) and 23.7% (*n* = 3,204) for men. Approximately 2 years after diagnosis, PD-related complications (pneumonia, orthopedic injuries, and PD-related hospitalization) occurred for the first time (women: 1.8 years; men: 2.3 years), and after 5 years, 72.9% (*n* = 6,397) of women, and 68.7% (*n* = 9,287) of men had experienced at least one.

**Discussion:** Considering the strengths and limitations of our methods, our findings suggest that women experience complications and access most healthcare services sooner after diagnosis and more frequently than men. The identified sex differences extend the debate about phenotypical differences in PD between men and women.

## Introduction

During the course of the disease, a patient with Parkinson's disease (PD) visits many different healthcare providers from different disciplines (1). This “journey through the healthcare system” varies per individual because of heterogeneity of symptoms, differences in disease progression rate, and the occurrence of PD-related complications. One important source of this variation might be sex differences in the presentation of PD (2). For example, numerous studies confirm that the incidence, and prevalence of PD is higher in men (2–6), that the disease starts at an earlier age in men (2, 7) and that the disease progresses faster in men (7, 8). In women, PD tends to be more often tremor-dominant (2, 7, 9), while in men it is more often the akinetic-rigid type (2, 10, 11).

We do not know if these sex differences translate to different patient journeys between men and women with PD. But when striving for optimal patient-centered and integrated care, it is vital to understand what the patient journeys look like. As shown for other diseases (12–15) such insights can be used to improve access and optimize coordination of care. In this paper, we use medical claims data to reconstruct the sex-specific journey for Dutch PD patients during the first 5 years after diagnosis.

In the Netherlands, the patient's journey starts when a general practitioner makes a referral to a neurologist when symptoms of Parkinson's appear. Neurologists, all located in hospitals, make the diagnosis. Thereafter, a PD patient visits the hospital every 3 months, to see their neurologist, who is supported by nurses or nurse specialists. The nurse and neurologist work in close collaboration with allied healthcare professionals in the community, including, e.g., physiotherapists, occupational therapists and speech and language therapists. Hospital care is covered by the compulsory health insurance, whereas allied healthcare services are covered by additional insurance package, which is not compulsory but taken up by over 80% of the Dutch people. In addition, the Netherlands stands out with comparatively low out-of-pocket payments (16). This probably reduces any possible selection bias due to differences in price responsiveness among PD patients. In the analysis, we therefore focus on the most frequently involved healthcare disciplines (neurologists, allied healthcare therapists, and general practitioners) and on recognized clinical milestones (PD-related complications, nursing home admission, and death).

## Materials and Methods

To reconstruct the PD patient journey, we used medical claims data of all PD patients diagnosed between 2012 and 2016 in the Netherlands. The dataset was made available through Vektis, a not-for-profit organization that combines claims data of all Dutch healthcare insurance companies (17). Since all Dutch citizens are obliged by law to have a healthcare insurance, the Vektis database contains the claims data of ~99.8% of the Dutch population (18) [17.3 million people (19)]. The claims database contains data on primary care, emergency care and hospital care, plus nursing home residency. The dataset was anonymized by Vektis, making available only the sex, year of birth, and a unique random identifier for each individual. The key to the identifiers was not available to the researchers.

Similar to a recent paper using similar Dutch claims data in PD (20), we included only patients who had at least one diagnosis-related group code (DRG code) for PD. In the Netherlands, PD can only be diagnosed by a neurologist. We therefore regarded the first PD-related neurology DRG as the moment of diagnosis and, as such, as the starting point of the journey.

To reconstruct the patient journey, we included the professionals most frequently involved in the treatment of PD. These are neurologists (together with specialized PD nurses, since both claim their activities under the DRG code of the hospital), physiotherapists, occupational therapists, speech and language therapists, and general practitioners. For every included claim, we calculated how many days after the first diagnosis the activity had occurred. Next, we selected the 1st, 10, 20, and 30th visit to the general practitioner and the allied healthcare therapists. Unlike these disciplines, claims related to hospital care are defined in a DRG model, rather than by a pay-per-visit model. The first PD-related DRG includes at least one visit to a neurologist, but the actual number of visits may be higher. A subsequent PD-DRG can only start 90 days after the initial PD-DRG, and contains at least one visit to a neurologist or specialized PD nurse. Third and subsequent PD-DRGs can only start 365 days after the previous one. Consequently, the maximum number of PD-DRGs within the first 5 years after diagnosis is six. We therefore selected the first six PD-DRGs to assess utilization of neurologist. In a similar way, we identified the time after diagnosis until five clinical milestones in the patient's journey had been reached, using a methodology previously used in a comparable analysis: (21) nursing home admission, hospitalization for three PD-related complications (unexpected hospitalization for PD, pneumonia, orthopedic injuries) (20, 22) and, finally, death.

We used event history analysis with Kaplan-Meyer estimators to determine after how many days the average patient received specific care or reached a clinical milestone. This method deals with differences in length of follow-up between patients. The follow-up length was calculated for every patient as the number of days from diagnosis till death or till December 31st 2016, i.e., the last data point in the dataset. The median values of the time-to-event analysis were plotted on a timeline, representing the journey of the average PD patient. We constructed one timeline for men, and one for women. We chose median values over mean values because of the considerable differences in length of follow-up in our sample. Sex differences were statistically analyzed using log rank tests.

### Standard Protocol Approvals, Registrations, and Patient Consents

This study was approved by the institutional review board of the Radboud University Medical Center with a waiver of consent for participants in the study.

### Data Availability Policy

All data, published or not published within the article, is accessible through Vektis. Analyses were performed in SAS.

## Results

We included medical claims data of all 22,293 newly diagnosed patients in the analyses. As shown in Table 1, the population consists mainly of elderly individuals, a minority of whom lives in a nursing home before diagnosis.

**Table 1:** General characteristics of the population (*n* = 22,293).

	**Men** **(*n* = 13,518; 60.6%)**	**Women** **(*n* = 8,775; 39.4%)**
Age at diagnosis (mean, in years)	71.6 (SD: 9.9)	72.5 (SD: 10.2)
Early onset PD [<50 years at diagnosis, *n* (%)]	421 (3.1%)	278 (3.2%)
Length of follow-up (mean)	2.5 years (SD: 1.4)	2.5 years (SD: 1.4)
Living in a nursing home at time of diagnosis [*n* (%)]	673 (5.0%)	729 (8.3%)
Death during follow-up [*n* (%)]	2,469 (18.3%)	1,284 (14.6%)

### Healthcare Utilization

**Figure 1:**
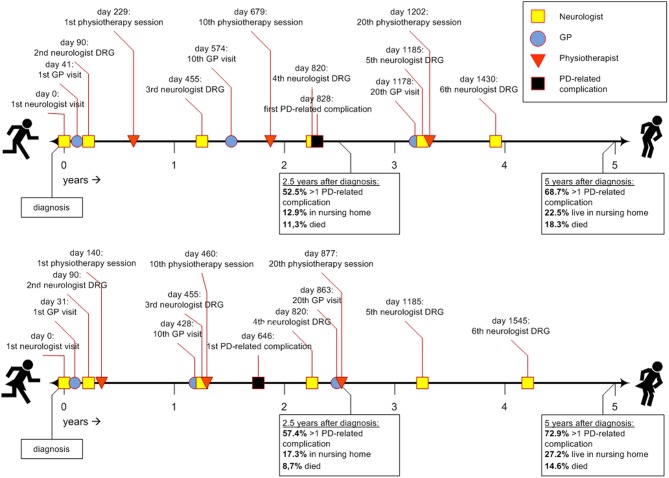
Timeline of the average Parkinson's patient journey during the first 5 years after diagnosis. DRG, diagnosis related group; GP, general practitioner; PD, Parkinson's disease.

Figure 1 shows two timelines representing the sex-specific patient journey of men and women with PD. Table 2 shows the inter quartile ranges (IQRs).

**Table 2:** Time (in days, since diagnosis) till relevant provider contacts and clinical milestones during the patient journey, by sex.

	**Women median (1st−3rd quartile)**	**Men median (1st−3rd quartile)**	***P*-value**
**PROVIDER CONTACT**			
1st neurology DRG	0 (0–0)	0 (0–0)	0.270
2nd neurology DRG	90 (90–240)	90 (90–210)	0.079
3rd neurology DRG	455 (360–>1,826)	455 (330–1,454)	0.040
4th neurology DRG	820 (575–>1,826)	820 (575–>1,826)	0.280
5th neurology DRG	1,185 (935–>1,826)	1,185 (935–>1,826)	0.348
6th neurology DRG	1,545 (1,180–>1,826)	1,430 (1,180–>1,826)	0.333
1st general practitioner visit	31 (8–87)	41 (11–116)	<0.001
10th general practitioner visit	428 (231–824)	574 (301–1,112)	<0.001
20th general practitioner visit	863 (505–1,605)	1,178 (659–>1,826)	<0.001
30th general practitioner visit	>1,826 (830–>1,826)	>1,826 (1,082–>1,826)	<0.001
1st physiotherapist visit	140 (16–826)	229 (38–1,303)	<0.001
10th physiotherapist visit	460 (136–>1,826)	679 (177–>1,826)	<0.001
20th physiotherapist visit	877 (268–>1,826)	1,202 (341–>1,826)	<0.001
30th physiotherapist visit	>1,826 (415–>1,826)	>1,826 (530–>1,826)	<0.001
1st occupational therapist visit	>1,826 (986–>1,826)	>1,826 (1247–>1,826)	<0.001
1st speech and language therapist visit	>1,826 (>1,826–>1,826)	>1,826 (>1,826–>1,826)	<0.001
**CLINICAL MILESTONES**			
Nursing home admission	>1,826 (1,571–>1,826)	>1,826 (>1,826–>1,826)	<0.001
**1ST PD-RELATED COMPLICATION**			
Pneumonia	>1,826 (661–>1,826)	>1,826 (686–>1,826)	0.879
Orthopedic injuries	1114 (347–>1,826)	>1,826 (527–>1,826)	<0.001
Hospitalization	>1826 (>1,826–>1,826)	>1,826 (>1,826–>1,826)	0.359
All PD-related complications	646 (179–>1,826)	828 (214–>1,826)	<0.001
Death	>1,826 (>1,826–>1,826)	>1,826 (>1,826–>1,826)	<0.001

Approximately 1 month after diagnosis, patients first visited their general practitioner (women after 31 days; men after 41). Thereafter, women saw their general practitioner approximately once every 6 weeks (43 days). Men saw their general practitioner less often: approximately once every 8 to 9 weeks (59 days). For both sexes the frequency declined after the 20th visit (median >5 years for 30th visit). In all analyses, women visited their general practitioner significantly earlier than men (*p*-values < 0.001).

Three months after diagnosis, many patients saw their neurologist or specialized PD nurse again (both for men and women median = 90 days). However, a substantial part of the population also visited their neurologist or specialized PD nurse much later for the second time, i.e., not before 8 to 9 months after diagnosis [75th quartile value = 210 days (men) and 240 days (women)]. After the second visit, the frequency of claimed neurology DRGs was about once a year for both sexes, i.e., they visited a neurologist or specialized PD nurse at least once a year. Except for the third visit, where men used neurologist services slightly earlier than women, no significant sex differences were found.

Women with PD started their physiotherapy treatment ~5 months after diagnosis (median = 140 days). Their first 20 physiotherapy sessions took place about once every 5 to 6 weeks. Men started to visit a physiotherapist later after the diagnosis then women: 8 months after diagnosis (median = 229 days), and with a lower frequency: once every seven to 8 weeks. For both sexes the frequency declined after the 20th session (median >5 years for 30th physiotherapy sessions). In all analyses, women used physiotherapist services significantly earlier and within a shorter timespan than men (*p*-values < 0.001).

For occupational therapists and speech and language therapists, the median values for the first visits were not reached within the follow-up time of 5 years. Therefore, they are not displayed in Figure 1. After 5 years, 37.9% of the women (*n* = 3,326) and 33.1% of the men (*n* = 4,474) had visited an occupational therapist at least once. This was 18.5% for women (*n* = 1,623) and 23.7% for men (*n* = 3,204) for the first visit to speech and language therapist. These differences were statistically significant (*p*-values < 0.001).

### Clinical Milestones

**Figure 2:**
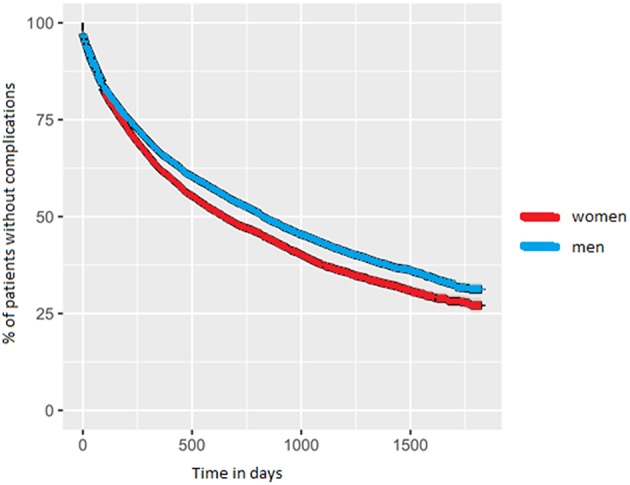
Kaplan-Meyer curve of the time-to-event analysis for PD-related complications.

Approximately 2 years after diagnosis, the first PD-related complication occurred. For women the median value was 1.8 years (IQR = 0.5–>5 years); for men this was 2.3 years (IQR = 0.6–>5 years; *p*-value < 0.001). We added the Kaplan-Meyer curve of this analysis in Figure 2. As shown in Table 2, orthopedic injuries were the most common complication, and occurred earlier in the course of the disease in women (*p*-value < 0.001). Five years after diagnosis, the percentage of patients that had experienced at least one PD-related complication was 72.9% in women (*n* = 6,397) and 68.7% in men (*n* = 9,287). The percentage of women admitted to a nursing home rose from 8.3% (*n* = 728) before diagnosis to 27.5% (*n* = 2,413) after 5 years of PD. In men this increase is from 5.0% (*n* = 676) to 22.5% (*n* = 3,042; *p* < 0.001). During the first 5 years after diagnosis, significantly fewer women died (14.6%) than men (18.3%, *p*-value = < 0.001).

## Discussion

The reconstruction of the Parkinson patient's journey through the Dutch healthcare sector during the first 5 years after diagnosis, reveals quantitative information about healthcare utilization and the occurrence of clinical milestones over time. It also reveals sex differences: in the Netherlands, women visit most of the included healthcare professionals sooner after diagnosis and more frequently. In addition, PD-related complications occur earlier in women than in men. A sizeable subgroup of patients is admitted to nursing homes within 5 years after diagnosis. Again, this happens more frequently in women. Finally, 14.6% of the women and 18.3% of the men died within 5 years after the diagnosis.

### Relation With Previous Findings

Our findings confirm and extend earlier work, from both inside and outside the Netherlands. The characteristics of our population are comparable to earlier work when it comes to general incidence (23) and the male predomination of the disease (2–6). Comparable values for age at diagnosis and percentage of early-onset PD were also found before (24). However, while most studies found a faster disease progression in men (7, 8), our findings suggest a more rapid disease progression in women, since they visited their healthcare professionals sooner and experienced orthopedic injuries earlier and more often after diagnosis. However, there might be other explanations for this observation. First, the included women were living relatively more often in a nursing home before diagnosis, indicating that they were probably in a worse physical condition at the outset. This might make them more prone to develop complications and also explain the more intense healthcare utilization. Patients living in nursing homes might also have easier access to the in-house allied health therapists. Second, the findings can indicate a doctor or patient delay in the diagnostic process in women, meaning that women receive the diagnosis relatively late in the course of the disease. This has been found earlier (25). Alternatively, women might find their way to healthcare professionals more effectively (or faster). This has been observed for other diseases (26, 27) but not previously for PD.

What surprised us was the high mortality rate and that patients experienced their first PD-related complication already 2 years after the diagnosis. No PD-specific literature is available to compare these results with. The average age at onset of 72 years is in line with other population-based cohort studies (28–30). The finding that 5.08.3% of the patients are living in a nursing home at diagnosis, which are relatively high, can be understood when considering that the Netherlands has relatively one of the largest long term care sectors in the world (31). Given that patients living in a nursing home are likely more vulnerable, this might explain the mortality and complication rates.

### Strengths and Limitations

Our methods have strengths and limitations. An important strength is that our dataset contained all newly diagnosed patients in the country over a period of 5 consecutive years. This reduces a potential selection bias. However, some selection may have resulted from our inclusion criteria. Since we included all patients with a first PD DRG, there might be some cases where the initial diagnosis of PD was wrong. PD is hard to diagnose, with reported diagnostic error rates of >10% (32). Therefore, we cannot exclude that some patients had other conditions, in particular one of the forms of atypical parkinsonism (23). Our sample most likely included people who were incorrectly diagnosed. A review of the literature, including 11 studies, concluded that the validity of clinical diagnosis of PD is not satisfying, which was the case for both non-experts and movement disorder specialists (33). We were not able to correct for this error in our analysis. However, since our population characteristics matched well with previous reports, we do not think that all these factors affected our data on a large scale. Moreover, it is unlikely that this diagnostic error affected men and women differentially. Another strength is that our study is based on highly standardized claims data. And we only used items that, although self-reported by healthcare professionals, are known to be reliably completed (34).

As claims data don't include detailed information about the clinical status of the patients, we were not able to correct for factors that confound and/or modify the relationship between sex and complications (35). This holds particularly true for co-morbidity and PD-related complications that are more frequently associated to female sex (e.g., dyskinesia, motor, and non-motor complications) (11). Also, the sex-difference in the occurrence of orthopedic injuries might be explained by the female predominance in osteoporosis. Therefore, our findings require confirmation in other, independent datasets.

Finally, our findings might be difficult to extrapolate to other countries with another organization of the healthcare system. For example, duration of visits and treatment intensity can differ between countries. Moreover, the presence of ParkinsonNet in the Netherlands contributed to the quality and role of allied health care professionals, and stimulated access to specialized and multidisciplinary Parkinson care (36).

### Practical Implications

Comparable work on other diseases suggests that our reconstruction of the patient journey may lead to better patient-centered care delivery. Specifically, it provides healthcare professionals an overview of where and when particular physicians get involved, which might reveal errors in providers' perspectives (37). It might act as a useful tool to gain insight in patient experiences (12, 37), to reveal barriers to access (13, 38), to detect gaps in care delivery (39, 40), and to improve coordination and quality of care (38, 41). The identified sex differences might contribute to the debate about differences in PD between men and women, extending earlier work on different phenotypes to now include contrasts in healthcare utilization as well. We hope these insights can lead to better and more personalized care for PD patients of both sexes.

## Data Availability

The datasets generated for this study are available on request to the corresponding author.

## Author Contributions

All authors contributed to the development of research questions and the selection and adaptation of the methods. FV, YM, and JK performed the analyses at Vektis. MT, AG, PJ, SO-P, MMu, BB, and MJM contributed in giving feedback during the writing process.

### Conflict of Interest Statement

BB currently serves as Associate Editor for the Journal of Parkinson's disease, serves on the editorial of Practical Neurology and Digital Biomarkers, has received honoraria from serving on the scientific advisory board for Abbvie, Biogen, UCB and Walk with Path, has received fees for speaking at conferences from AbbVie, Zambon, Roche, GE Healthcare and Bial, and has received research support from the Netherlands Organization for Scientific Research, the Michael J Fox Foundation, UCB, Abbvie, the Stichting Parkinson Fonds, the Hersenstichting Nederland, the Parkinson's Foundation, Verily Life Sciences, Horizon 2020, the Topsector Life Sciences and Health, and the Parkinson Vereniging.

The remaining authors declare that the research was conducted in the absence of any commercial or financial relationships that could be construed as a potential conflict of interest.
